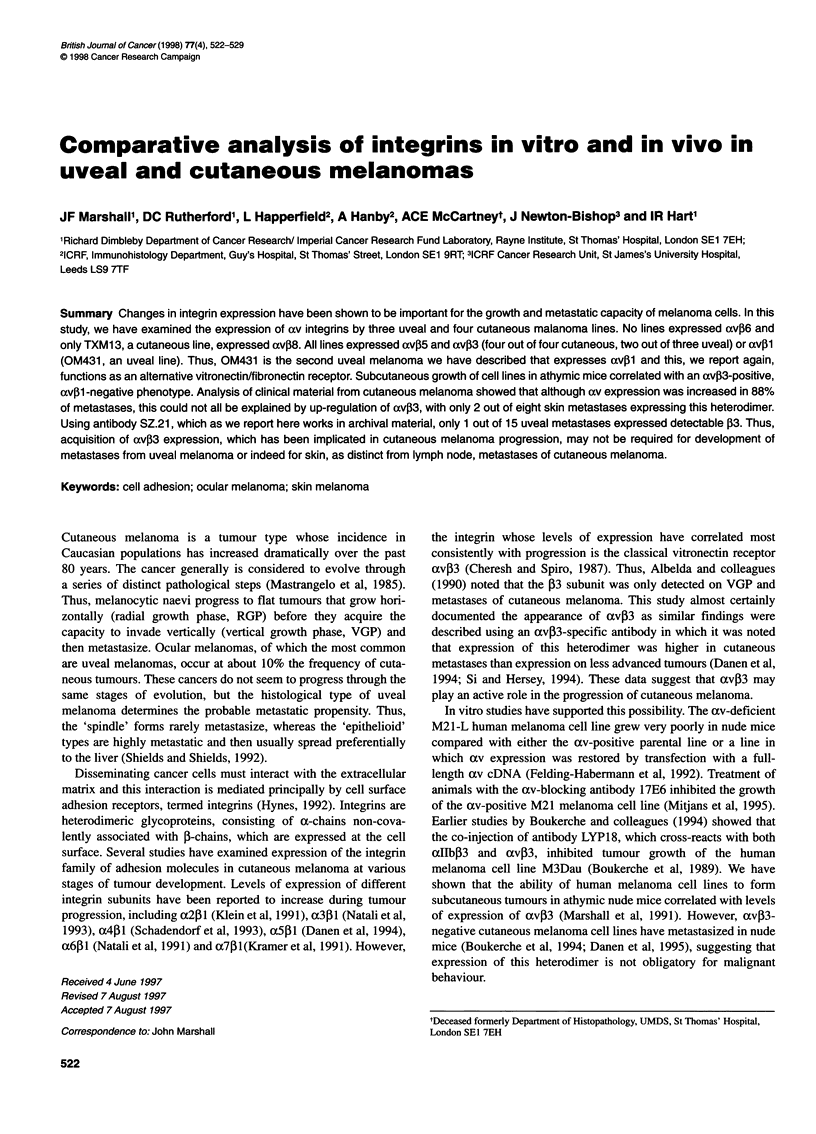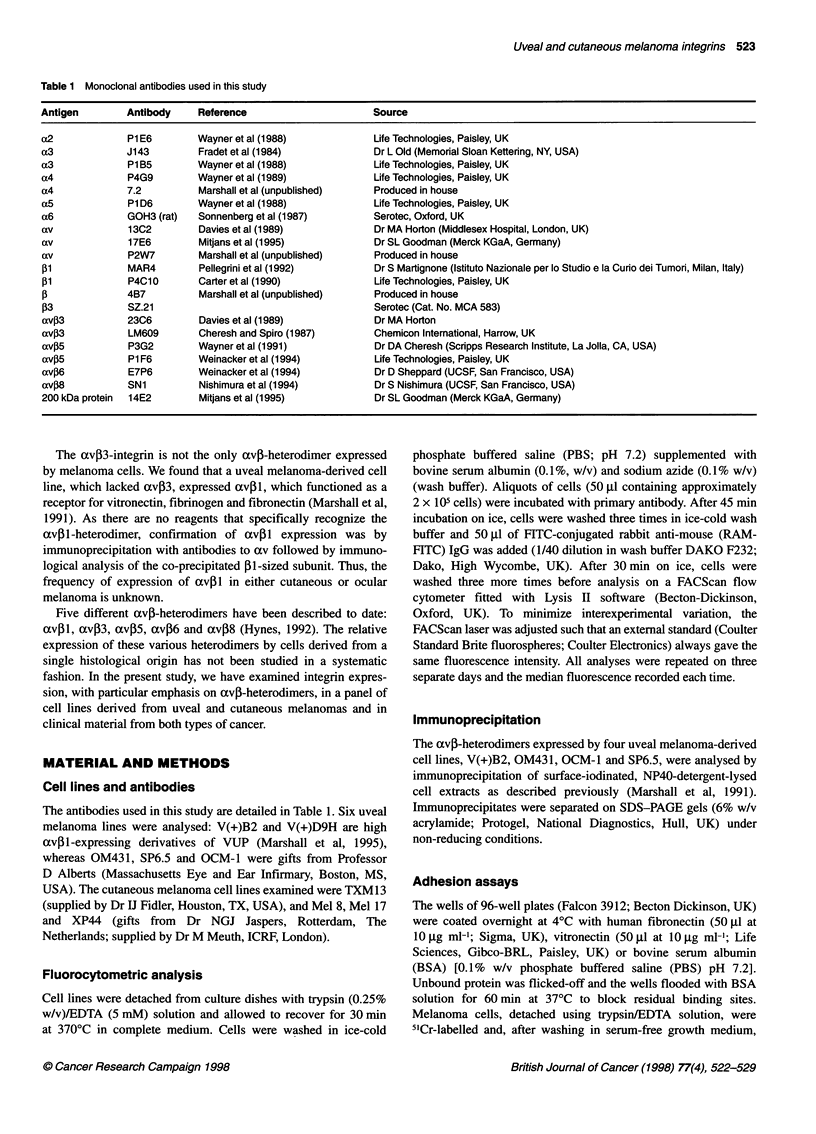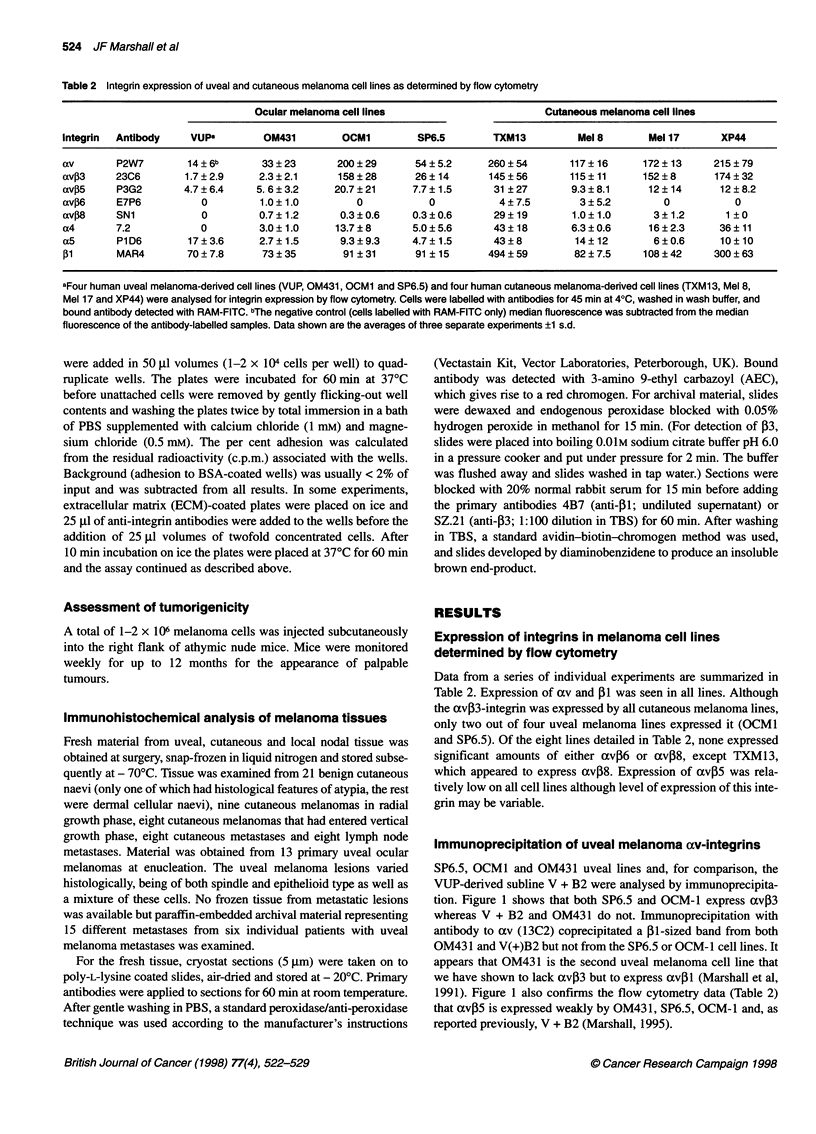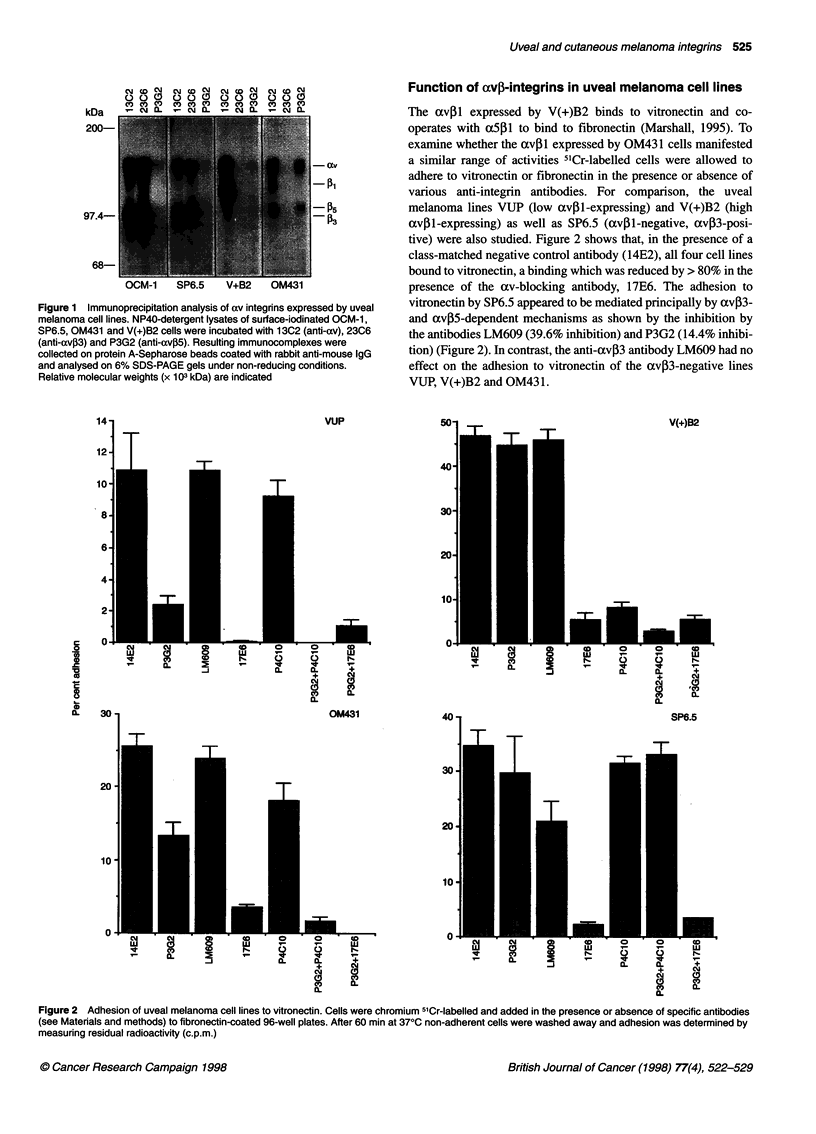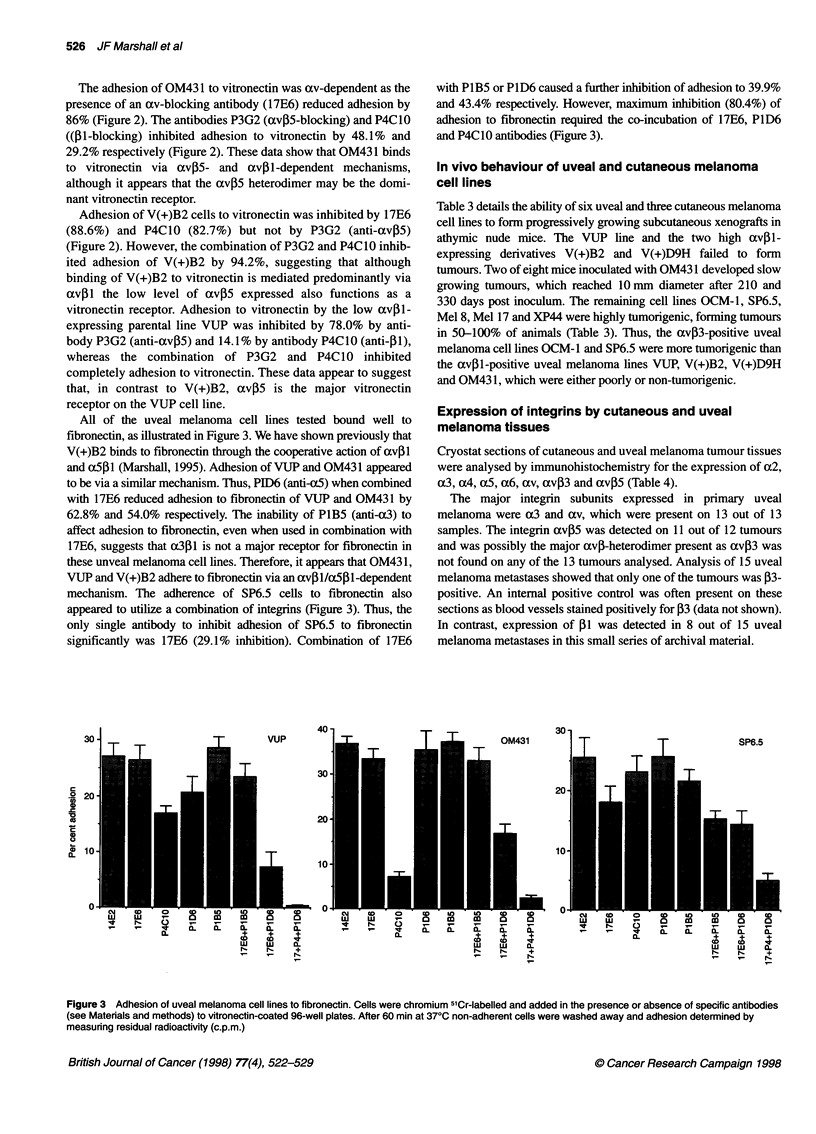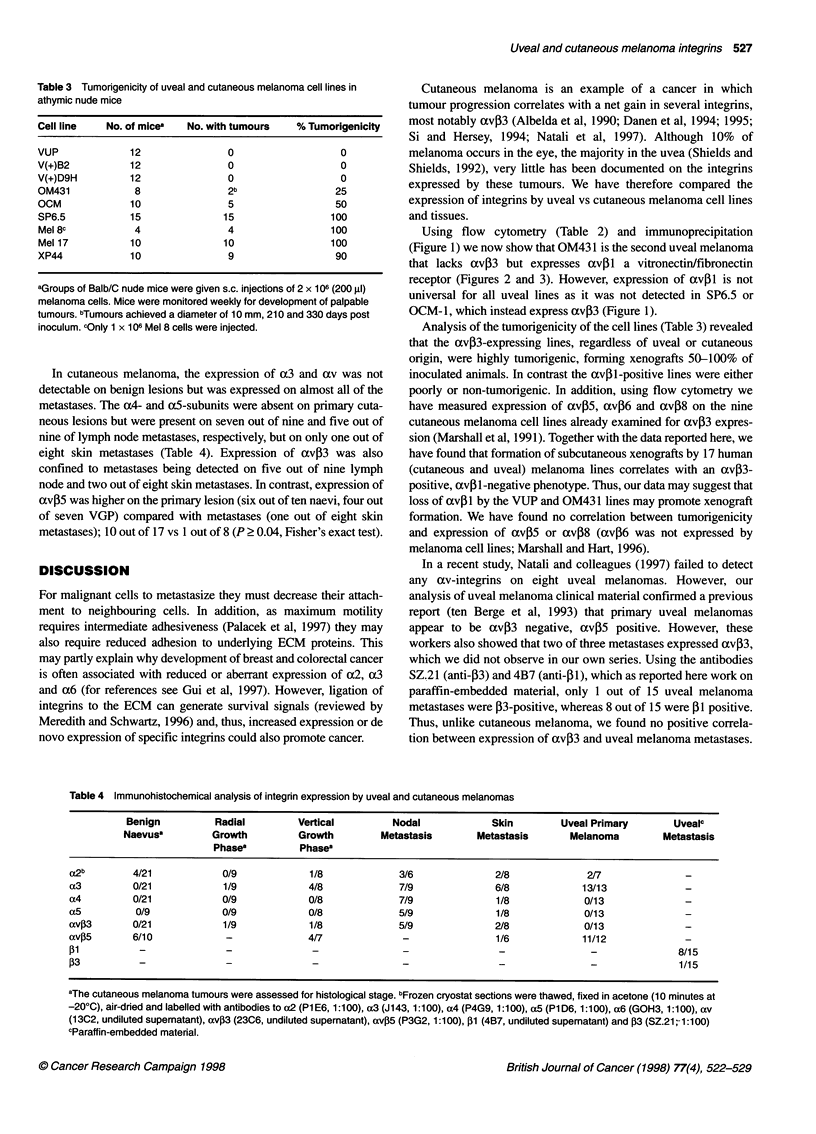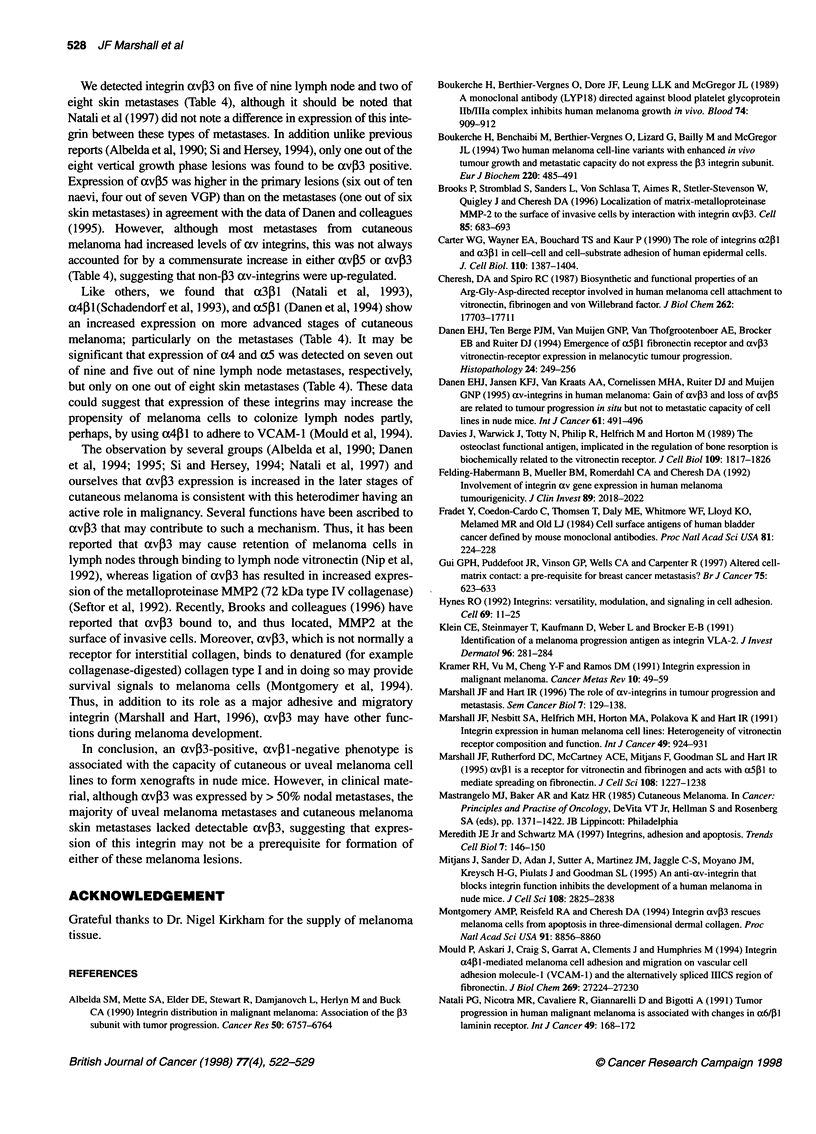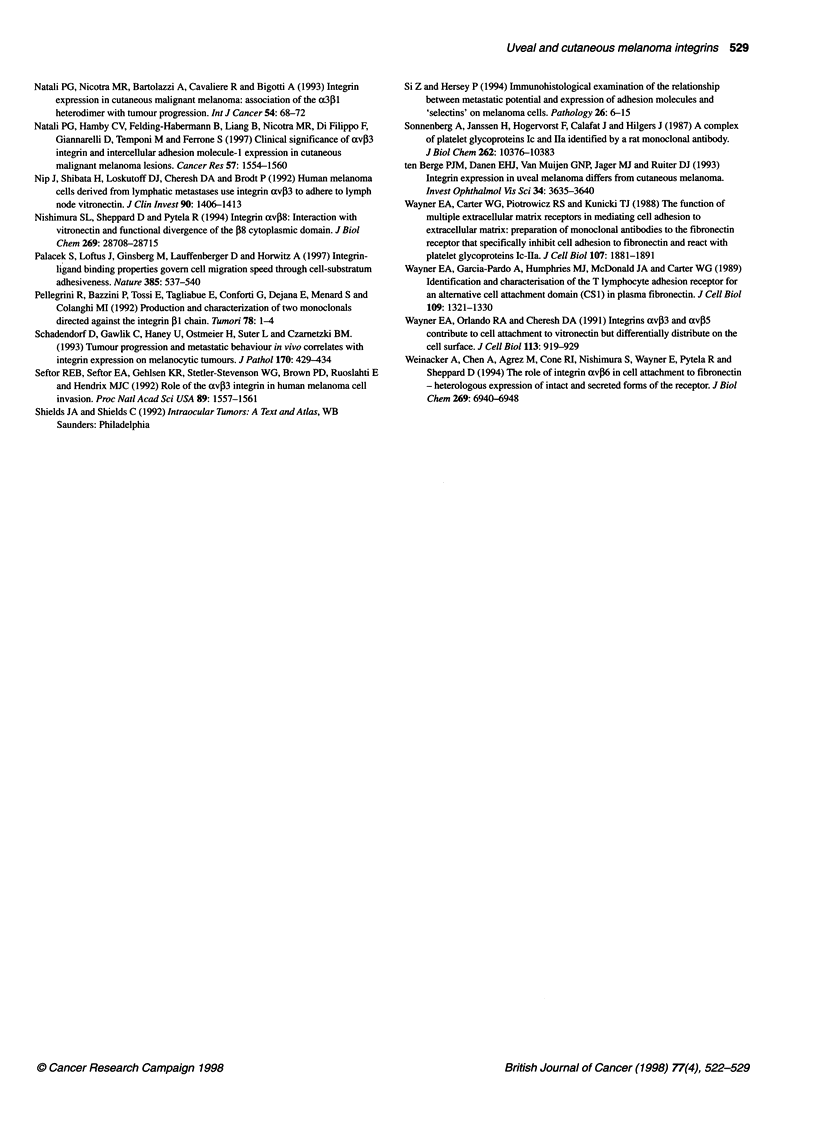# Comparative analysis of integrins in vitro and in vivo in uveal and cutaneous melanomas.

**DOI:** 10.1038/bjc.1998.85

**Published:** 1998-02

**Authors:** J. F. Marshall, D. C. Rutherford, L. Happerfield, A. Hanby, A. C. McCartney, J. Newton-Bishop, I. R. Hart

**Affiliations:** Richard Dimbleby Department of Cancer Research, Rayne Institute, St Thomas' Hospital, London.

## Abstract

**Images:**


British Journal of Cancer (1998) 77(4), 522–529
© 1998 Cancer Research Campaign

### Comparative analysis of integrins in vitro and in vivo in uveal and cutaneous melanomas

**JF Marshall[1], DC Rutherford[1], L Happerfield[2], A Hanby[2], ACE McCartney[†], J Newton-Bishop[3] and IR Hart[1]**

[1]Richard Dimbleby Department of Cancer Research/ Imperial Cancer Research Fund Laboratory, Rayne Institute, St Thomas' Hospital, London SE1 7EH; [2]ICRF, Immunohistology Department, Guy's Hospital, St Thomas' Street, London SE1 9RT; [3]ICRF Cancer Research Unit, St James's University Hospital, Leeds LS9 7TF

**Summary** Changes in integrin expression have been shown to be important for the growth and metastatic capacity of melanoma cells. In this study, we have examined the expression of αv integrins by three uveal and four cutaneous malanoma lines. No lines expressed αvβ6 and only TXM13, a cutaneous line, expressed αvβ8. All lines expressed αvβ5 and αvβ3 (four out of four cutaneous, two out of three uveal) or αvβ1 (OM431, an uveal line). Thus, OM431 is the second uveal melanoma we have described that expresses αvβ1 and this, we report again, functions as an alternative vitronectin/fibronectin receptor. Subcutaneous growth of cell lines in athymic mice correlated with an αvβ3-positive, αvβ1-negative phenotype. Analysis of clinical material from cutaneous melanoma showed that although αv expression was increased in 88% of metastases, this could not all be explained by up-regulation of αvβ3, with only 2 out of eight skin metastases expressing this heterodimer. Using antibody SZ.21, which as we report here works in archival material, only 1 out of 15 uveal metastases expressed detectable β3. Thus, acquisition of αvβ3 expression, which has been implicated in cutaneous melanoma progression, may not be required for development of metastases from uveal melanoma or indeed for skin, as distinct from lymph node, metastases of cutaneous melanoma.

**Keywords:** cell adhesion; ocular melanoma; skin melanoma

Cutaneous melanoma is a tumour type whose incidence in Caucasian populations has increased dramatically over the past 80 years. The cancer generally is considered to evolve through a series of distinct pathological steps (Mastrangelo et al, 1985). Thus, melanocytic naevi progress to flat tumours that grow horizontally (radial growth phase, RGP) before they acquire the capacity to invade vertically (vertical growth phase, VGP) and then metastasize. Ocular melanomas, of which the most common are uveal melanomas, occur at about 10% the frequency of cutaneous tumours. These cancers do not seem to progress through the same stages of evolution, but the histological type of uveal melanoma determines the probable metastatic propensity. Thus, the 'spindle' forms rarely metastasize, whereas the 'epithelioid' types are highly metastatic and then usually spread preferentially to the liver (Shields and Shields, 1992).

Disseminating cancer cells must interact with the extracellular matrix and this interaction is mediated principally by cell surface adhesion receptors, termed integrins (Hynes, 1992). Integrins are heterodimeric glycoproteins, consisting of α-chains non-covalently associated with β-chains, which are expressed at the cell surface. Several studies have examined expression of the integrin family of adhesion molecules in cutaneous melanoma at various stages of tumour development. Levels of expression of different integrin subunits have been reported to increase during tumour progression, including α2β1 (Klein et al, 1991), α3β1 (Natali et al, 1993), α4β1 (Schadendorf et al, 1993), α5β1 (Danen et al, 1994), α6β1 (Natali et al, 1991) and α7β1(Kramer et al, 1991). However, the integrin whose levels of expression have correlated most consistently with progression is the classical vitronectin receptor αvβ3 (Cheresh and Spiro, 1987). Thus, Albelda and colleagues (1990) noted that the β3 subunit was only detected on VGP and metastases of cutaneous melanoma. This study almost certainly documented the appearance of αvβ3 as similar findings were described using an αvβ3-specific antibody in which it was noted that expression of this heterodimer was higher in cutaneous metastases than expression on less advanced tumours (Danen et al, 1994; Si and Hersey, 1994). These data suggest that αvβ3 may play an active role in the progression of cutaneous melanoma.

In vitro studies have supported this possibility. The αv-deficient M21-L human melanoma cell line grew very poorly in nude mice compared with either the αv-positive parental line or a line in which αv expression was restored by transfection with a full-length αv cDNA (Felding-Habermann et al, 1992). Treatment of animals with the αv-blocking antibody 17E6 inhibited the growth of the αv-positive M21 melanoma cell line (Mitjans et al, 1995). Earlier studies by Boukerche and colleagues (1994) showed that the co-injection of antibody LYP18, which cross-reacts with both αIIbβ3 and αvβ3, inhibited tumour growth of the human melanoma cell line M3Dau (Boukerche et al, 1989). We have shown that the ability of human melanoma cell lines to form subcutaneous tumours in athymic nude mice correlated with levels of expression of αvβ3 (Marshall et al, 1991). However, αvβ3-negative cutaneous melanoma cell lines have metastasized in nude mice (Boukerche et al, 1994; Danen et al, 1995), suggesting that expression of this heterodimer is not obligatory for malignant behaviour.

*Received 4 June 1997*
*Revised 7 August 1997*
*Accepted 7 August 1997*

*Correspondence to:* John Marshall

[†]Deceased formerly Department of Histopathology, UMDS, St Thomas' Hospital, London SE1 7EH

**Table 1** Monoclonal antibodies used in this study

| Antigen | Antibody | Reference | Source |
|---|---|---|---|
| α2 | P1E6 | Wayner et al (1988) | Life Technologies, Paisley, UK |
| α3 | J143 | Fradet et al (1984) | Dr L Old (Memorial Sloan Kettering, NY, USA) |
| α3 | P1B5 | Wayner et al (1988) | Life Technologies, Paisley, UK |
| α4 | P4G9 | Wayner et al (1989) | Life Technologies, Paisley, UK |
| α4 | 7.2 | Marshall et al (unpublished) | Produced in house |
| α5 | P1D6 | Wayner et al (1988) | Life Technologies, Paisley, UK |
| α6 | GOH3 (rat) | Sonnenberg et al (1987) | Serotec, Oxford, UK |
| αv | 13C2 | Davies et al (1989) | Dr MA Horton (Middlesex Hospital, London, UK) |
| αv | 17E6 | Mitjans et al (1995) | Dr SL Goodman (Merck KGaA, Germany) |
| αv | P2W7 | Marshall et al (unpublished) | Produced in house |
| β1 | MAR4 | Pellegrini et al (1992) | Dr S Martignone (Istituto Nazionale per lo Studio e la Curio dei Tumori, Milan, Italy) |
| β1 | P4C10 | Carter et al (1990) | Life Technologies, Paisley, UK |
| β | 4B7 | Marshall et al (unpublished) | Produced in house |
| β3 | SZ.21 | | Serotec (Cat. No. MCA 583) |
| αvβ3 | 23C6 | Davies et al (1989) | Dr MA Horton |
| αvβ3 | LM609 | Cheresh and Spiro (1987) | Chemicon International, Harrow, UK |
| αvβ5 | P3G2 | Wayner et al (1991) | Dr DA Cheresh (Scripps Research Institute, La Jolla, CA, USA) |
| αvβ5 | P1F6 | Weinacker et al (1994) | Life Technologies, Paisley, UK |
| αvβ6 | E7P6 | Weinacker et al (1994) | Dr D Sheppard (UCSF, San Francisco, USA) |
| αvβ8 | SN1 | Nishimura et al (1994) | Dr S Nishimura (UCSF, San Francisco, USA) |
| 200 kDa protein | 14E2 | Mitjans et al (1995) | Dr SL Goodman (Merck KGaA, Germany) |

The αvβ3-integrin is not the only αvβ-heterodimer expressed by melanoma cells. We found that a uveal melanoma-derived cell line, which lacked αvβ3, expressed αvβ1, which functioned as a receptor for vitronectin, fibrinogen and fibronectin (Marshall et al, 1991). As there are no reagents that specifically recognize the αvβ1-heterodimer, confirmation of αvβ1 expression was by immunoprecipitation with antibodies to αv followed by immunological analysis of the co-precipitated β1-sized subunit. Thus, the frequency of expression of αvβ1 in either cutaneous or ocular melanoma is unknown.

Five different αvβ-heterodimers have been described to date: αvβ1, αvβ3, αvβ5, αvβ6 and αvβ8 (Hynes, 1992). The relative expression of these various heterodimers by cells derived from a single histological origin has not been studied in a systematic fashion. In the present study, we have examined integrin expression, with particular emphasis on αvβ-heterodimers, in a panel of cell lines derived from uveal and cutaneous melanomas and in clinical material from both types of cancer.

### MATERIAL AND METHODS

### Cell lines and antibodies

The antibodies used in this study are detailed in Table 1. Six uveal melanoma lines were analysed: V(+)B2 and V(+)D9H are high αvβ1-expressing derivatives of VUP (Marshall et al, 1995), whereas OM431, SP6.5 and OCM-1 were gifts from Professor D Alberts (Massachusetts Eye and Ear Infirmary, Boston, MS, USA). The cutaneous melanoma cell lines examined were TXM13 (supplied by Dr IJ Fidler, Houston, TX, USA), and Mel 8, Mel 17 and XP44 (gifts from Dr NGJ Jaspers, Rotterdam, The Netherlands; supplied by Dr M Meuth, ICRF, London).

### Fluorocytometric analysis

Cell lines were detached from culture dishes with trypsin (0.25% w/v)/EDTA (5 mM) solution and allowed to recover for 30 min at 370°C in complete medium. Cells were washed in ice-cold phosphate buffered saline (PBS; pH 7.2) supplemented with bovine serum albumin (0.1%, w/v) and sodium azide (0.1% w/v) (wash buffer). Aliquots of cells (50 μl containing approximately $2 \times 10^5$ cells) were incubated with primary antibody. After 45 min incubation on ice, cells were washed three times in ice-cold wash buffer and 50 μl of FITC-conjugated rabbit anti-mouse (RAM-FITC) IgG was added (1/40 dilution in wash buffer DAKO F232; Dako, High Wycombe, UK). After 30 min on ice, cells were washed three more times before analysis on a FACScan flow cytometer fitted with Lysis II software (Becton-Dickinson, Oxford, UK). To minimize interexperimental variation, the FACScan laser was adjusted such that an external standard (Coulter Standard Brite fluorospheres; Coulter Electronics) always gave the same fluorescence intensity. All analyses were repeated on three separate days and the median fluorescence recorded each time.

### Immunoprecipitation

The αvβ-heterodimers expressed by four uveal melanoma-derived cell lines, V(+)B2, OM431, OCM-1 and SP6.5, were analysed by immunoprecipitation of surface-iodinated, NP40-detergent-lysed cell extracts as described previously (Marshall et al, 1991). Immunoprecipitates were separated on SDS–PAGE gels (6% w/v acrylamide; Protogel, National Diagnostics, Hull, UK) under non-reducing conditions.

### Adhesion assays

The wells of 96-well plates (Falcon 3912; Becton Dickinson, UK) were coated overnight at 4°C with human fibronectin (50 μl at 10 μg $ml^{-1}$; Sigma, UK), vitronectin (50 μl at 10 μg $ml^{-1}$; Life Sciences, Gibco-BRL, Paisley, UK) or bovine serum albumin (BSA) [0.1% w/v phosphate buffered saline (PBS) pH 7.2]. Unbound protein was flicked-off and the wells flooded with BSA solution for 60 min at 37°C to block residual binding sites. Melanoma cells, detached using trypsin/EDTA solution, were $^{51}Cr$-labelled and, after washing in serum-free growth medium,

© Cancer Research Campaign 1998

Table 2 Integrin expression of uveal and cutaneous melanoma cell lines as determined by flow cytometry

| Integrin | Antibody | Ocular melanoma cell lines | | | | Cutaneous melanoma cell lines | | | |
|---|---|---|---|---|---|---|---|---|---|
| | | VUP[a] | OM431 | OCM1 | SP6.5 | TXM13 | Mel 8 | Mel 17 | XP44 |
| αv | P2W7 | 14 ± 6[b] | 33 ± 23 | 200 ± 29 | 54 ± 5.2 | 260 ± 54 | 117 ± 16 | 172 ± 13 | 215 ± 79 |
| αvβ3 | 23C6 | 1.7 ± 2.9 | 2.3 ± 2.1 | 158 ± 28 | 26 ± 14 | 145 ± 56 | 115 ± 11 | 152 ± 8 | 174 ± 32 |
| αvβ5 | P3G2 | 4.7 ± 6.4 | 5. 6 ± 3.2 | 20.7 ± 21 | 7.7 ± 1.5 | 31 ± 27 | 9.3 ± 8.1 | 12 ± 14 | 12 ± 8.2 |
| αvβ6 | E7P6 | 0 | 1.0 ± 1.0 | 0 | 0 | 4 ± 7.5 | 3 ± 5.2 | 0 | 0 |
| αvβ8 | SN1 | 0 | 0.7 ± 1.2 | 0.3 ± 0.6 | 0.3 ± 0.6 | 29 ± 19 | 1.0 ± 1.0 | 3 ± 1.2 | 1 ± 0 |
| α4 | 7.2 | 0 | 3.0 ± 1.0 | 13.7 ± 8 | 5.0 ± 5.6 | 43 ± 18 | 6.3 ± 0.6 | 16 ± 2.3 | 36 ± 11 |
| α5 | P1D6 | 17 ± 3.6 | 2.7 ± 1.5 | 9.3 ± 9.3 | 4.7 ± 1.5 | 43 ± 8 | 14 ± 12 | 6 ± 0.6 | 10 ± 10 |
| β1 | MAR4 | 70 ± 7.8 | 73 ± 35 | 91 ± 31 | 91 ± 15 | 494 ± 59 | 82 ± 7.5 | 108 ± 42 | 300 ± 63 |

[a]Four human uveal melanoma-derived cell lines (VUP, OM431, OCM1 and SP6.5) and four human cutaneous melanoma-derived cell lines (TXM13, Mel 8, Mel 17 and XP44) were analysed for integrin expression by flow cytometry. Cells were labelled with antibodies for 45 min at 4°C, washed in wash buffer, and bound antibody detected with RAM-FITC. [b]The negative control (cells labelled with RAM-FITC only) median fluorescence was subtracted from the median fluorescence of the antibody-labelled samples. Data shown are the averages of three separate experiments ±1 s.d.

were added in 50 μl volumes (1–2 × $10^4$ cells per well) to quadruplicate wells. The plates were incubated for 60 min at 37°C before unattached cells were removed by gently flicking-out well contents and washing the plates twice by total immersion in a bath of PBS supplemented with calcium chloride (1 mM) and magnesium chloride (0.5 mM). The per cent adhesion was calculated from the residual radioactivity (c.p.m.) associated with the wells. Background (adhesion to BSA-coated wells) was usually < 2% of input and was subtracted from all results. In some experiments, extracellular matrix (ECM)-coated plates were placed on ice and 25 μl of anti-integrin antibodies were added to the wells before the addition of 25 μl volumes of twofold concentrated cells. After 10 min incubation on ice the plates were placed at 37°C for 60 min and the assay continued as described above.

### Assessment of tumorigenicity

A total of 1–2 × $10^6$ melanoma cells was injected subcutaneously into the right flank of athymic nude mice. Mice were monitored weekly for up to 12 months for the appearance of palpable tumours.

### Immunohistochemical analysis of melanoma tissues

Fresh material from uveal, cutaneous and local nodal tissue was obtained at surgery, snap-frozen in liquid nitrogen and stored subsequently at – 70°C. Tissue was examined from 21 benign cutaneous naevi (only one of which had histological features of atypia, the rest were dermal cellular naevi), nine cutaneous melanomas in radial growth phase, eight cutaneous melanomas that had entered vertical growth phase, eight cutaneous metastases and eight lymph node metastases. Material was obtained from 13 primary uveal ocular melanomas at enucleation. The uveal melanoma lesions varied histologically, being of both spindle and epithelioid type as well as a mixture of these cells. No frozen tissue from metastatic lesions was available but paraffin-embedded archival material representing 15 different metastases from six individual patients with uveal melanoma metastases was examined.

For the fresh tissue, cryostat sections (5 μm) were taken on to poly-L-lysine coated slides, air-dried and stored at – 20°C. Primary antibodies were applied to sections for 60 min at room temperature. After gentle washing in PBS, a standard peroxidase/anti-peroxidase technique was used according to the manufacturer's instructions (Vectastain Kit, Vector Laboratories, Peterborough, UK). Bound antibody was detected with 3-amino 9-ethyl carbazoyl (AEC), which gives rise to a red chromogen. For archival material, slides were dewaxed and endogenous peroxidase blocked with 0.05% hydrogen peroxide in methanol for 15 min. (For detection of β3, slides were placed into boiling 0.01M sodium citrate buffer pH 6.0 in a pressure cooker and put under pressure for 2 min. The buffer was flushed away and slides washed in tap water.) Sections were blocked with 20% normal rabbit serum for 15 min before adding the primary antibodies 4B7 (anti-β1; undiluted supernatant) or SZ.21 (anti-β3; 1:100 dilution in TBS) for 60 min. After washing in TBS, a standard avidin–biotin–chromogen method was used, and slides developed by diaminobenzidene to produce an insoluble brown end-product.

### RESULTS

### Expression of integrins in melanoma cell lines determined by flow cytometry

Data from a series of individual experiments are summarized in Table 2. Expression of αv and β1 was seen in all lines. Although the αvβ3-integrin was expressed by all cutaneous melanoma lines, only two out of four uveal melanoma lines expressed it (OCM1 and SP6.5). Of the eight lines detailed in Table 2, none expressed significant amounts of either αvβ6 or αvβ8, except TXM13, which appeared to express αvβ8. Expression of αvβ5 was relatively low on all cell lines although level of expression of this integrin may be variable.

### Immunoprecipitation of uveal melanoma αv-integrins

SP6.5, OCM1 and OM431 uveal lines and, for comparison, the VUP-derived subline V + B2 were analysed by immunoprecipitation. Figure 1 shows that both SP6.5 and OCM-1 express αvβ3 whereas V + B2 and OM431 do not. Immunoprecipitation with antibody to αv (13C2) coprecipitated a β1-sized band from both OM431 and V(+)B2 but not from the SP6.5 or OCM-1 cell lines. It appears that OM431 is the second uveal melanoma cell line that we have shown to lack αvβ3 but to express αvβ1 (Marshall et al, 1991). Figure 1 also confirms the flow cytometry data (Table 2) that αvβ5 is expressed weakly by OM431, SP6.5, OCM-1 and, as reported previously, V + B2 (Marshall, 1995).

 © Cancer Research Campaign 1998

**Figure 1** Immunoprecipitation analysis of αv integrins expressed by uveal melanoma cell lines. NP40-detergent lysates of surface-iodinated OCM-1, SP6.5, OM431 and V(+)B2 cells were incubated with 13C2 (anti-αv), 23C6 (anti-αvβ3) and P3G2 (anti-αvβ5). Resulting immunocomplexes were collected on protein A-Sepharose beads coated with rabbit anti-mouse IgG and analysed on 6% SDS-PAGE gels under non-reducing conditions. Relative molecular weights (× $10^3$ kDa) are indicated

### Function of αvβ-integrins in uveal melanoma cell lines

The αvβ1 expressed by V(+)B2 binds to vitronectin and co-operates with α5β1 to bind to fibronectin (Marshall, 1995). To examine whether the αvβ1 expressed by OM431 cells manifested a similar range of activities $^{51}$Cr-labelled cells were allowed to adhere to vitronectin or fibronectin in the presence or absence of various anti-integrin antibodies. For comparison, the uveal melanoma lines VUP (low αvβ1-expressing) and V(+)B2 (high αvβ1-expressing) as well as SP6.5 (αvβ1-negative, αvβ3-positive) were also studied. Figure 2 shows that, in the presence of a class-matched negative control antibody (14E2), all four cell lines bound to vitronectin, a binding which was reduced by > 80% in the presence of the αv-blocking antibody, 17E6. The adhesion to vitronectin by SP6.5 appeared to be mediated principally by αvβ3- and αvβ5-dependent mechanisms as shown by the inhibition by the antibodies LM609 (39.6% inhibition) and P3G2 (14.4% inhibition) (Figure 2). In contrast, the anti-αvβ3 antibody LM609 had no effect on the adhesion to vitronectin of the αvβ3-negative lines VUP, V(+)B2 and OM431.

**Figure 2** Adhesion of uveal melanoma cell lines to vitronectin. Cells were chromium $^{51}$Cr-labelled and added in the presence or absence of specific antibodies (see Materials and methods) to fibronectin-coated 96-well plates. After 60 min at 37°C non-adherent cells were washed away and adhesion was determined by measuring residual radioactivity (c.p.m.)

The adhesion of OM431 to vitronectin was αv-dependent as the presence of an αv-blocking antibody (17E6) reduced adhesion by 86% (Figure 2). The antibodies P3G2 (αvβ5-blocking) and P4C10 ((β1-blocking) inhibited adhesion to vitronectin by 48.1% and 29.2% respectively (Figure 2). These data show that OM431 binds to vitronectin via αvβ5- and αvβ1-dependent mechanisms, although it appears that the αvβ5 heterodimer may be the dominant vitronectin receptor.

Adhesion of V(+)B2 cells to vitronectin was inhibited by 17E6 (88.6%) and P4C10 (82.7%) but not by P3G2 (anti-αvβ5) (Figure 2). However, the combination of P3G2 and P4C10 inhibited adhesion of V(+)B2 by 94.2%, suggesting that although binding of V(+)B2 to vitronectin is mediated predominantly via αvβ1 the low level of αvβ5 expressed also functions as a vitronectin receptor. Adhesion to vitronectin by the low αvβ1-expressing parental line VUP was inhibited by 78.0% by antibody P3G2 (anti-αvβ5) and 14.1% by antibody P4C10 (anti-β1), whereas the combination of P3G2 and P4C10 inhibited completely adhesion to vitronectin. These data appear to suggest that, in contrast to V(+)B2, αvβ5 is the major vitronectin receptor on the VUP cell line.

All of the uveal melanoma cell lines tested bound well to fibronectin, as illustrated in Figure 3. We have shown previously that V(+)B2 binds to fibronectin through the cooperative action of αvβ1 and α5β1 (Marshall, 1995). Adhesion of VUP and OM431 appeared to be via a similar mechanism. Thus, PID6 (anti-α5) when combined with 17E6 reduced adhesion to fibronectin of VUP and OM431 by 62.8% and 54.0% respectively. The inability of P1B5 (anti-α3) to affect adhesion to fibronectin, even when used in combination with 17E6, suggests that α3β1 is not a major receptor for fibronectin in these unveal melanoma cell lines. Therefore, it appears that OM431, VUP and V(+)B2 adhere to fibronectin via an αvβ1/α5β1-dependent mechanism. The adherence of SP6.5 cells to fibronectin also appeared to utilize a combination of integrins (Figure 3). Thus, the only single antibody to inhibit adhesion of SP6.5 to fibronectin significantly was 17E6 (29.1% inhibition). Combination of 17E6 with P1B5 or P1D6 caused a further inhibition of adhesion to 39.9% and 43.4% respectively. However, maximum inhibition (80.4%) of adhesion to fibronectin required the co-incubation of 17E6, P1D6 and P4C10 antibodies (Figure 3).

### In vivo behaviour of uveal and cutaneous melanoma cell lines

Table 3 details the ability of six uveal and three cutaneous melanoma cell lines to form progressively growing subcutaneous xenografts in athymic nude mice. The VUP line and the two high αvβ1-expressing derivatives V(+)B2 and V(+)D9H failed to form tumours. Two of eight mice inoculated with OM431 developed slow growing tumours, which reached 10 mm diameter after 210 and 330 days post inoculum. The remaining cell lines OCM-1, SP6.5, Mel 8, Mel 17 and XP44 were highly tumorigenic, forming tumours in 50–100% of animals (Table 3). Thus, the αvβ3-positive uveal melanoma cell lines OCM-1 and SP6.5 were more tumorigenic than the αvβ1-positive uveal melanoma lines VUP, V(+)B2, V(+)D9H and OM431, which were either poorly or non-tumorigenic.

### Expression of integrins by cutaneous and uveal melanoma tissues

Cryostat sections of cutaneous and uveal melanoma tumour tissues were analysed by immunohistochemistry for the expression of α2, α3, α4, α5, α6, αv, αvβ3 and αvβ5 (Table 4).

The major integrin subunits expressed in primary uveal melanoma were α3 and αv, which were present on 13 out of 13 samples. The integrin αvβ5 was detected on 11 out of 12 tumours and was possibly the major αvβ-heterodimer present as αvβ3 was not found on any of the 13 tumours analysed. Analysis of 15 uveal melanoma metastases showed that only one of the tumours was β3-positive. An internal positive control was often present on these sections as blood vessels stained positively for β3 (data not shown). In contrast, expression of β1 was detected in 8 out of 15 uveal melanoma metastases in this small series of archival material.

**Figure 3** Adhesion of uveal melanoma cell lines to fibronectin. Cells were chromium $^{51}Cr$-labelled and added in the presence or absence of specific antibodies (see Materials and methods) to vitronectin-coated 96-well plates. After 60 min at 37°C non-adherent cells were washed away and adhesion determined by measuring residual radioactivity (c.p.m.)

 © Cancer Research Campaign 1998

**Table 3** Tumorigenicity of uveal and cutaneous melanoma cell lines in athymic nude mice

| Cell line | No. of mice[a] | No. with tumours | % Tumorigenicity |
|---|---|---|---|
| VUP | 12 | 0 | 0 |
| V(+)B2 | 12 | 0 | 0 |
| V(+)D9H | 12 | 0 | 0 |
| OM431 | 8 | 2[b] | 25 |
| OCM | 10 | 5 | 50 |
| SP6.5 | 15 | 15 | 100 |
| Mel 8[c] | 4 | 4 | 100 |
| Mel 17 | 10 | 10 | 100 |
| XP44 | 10 | 9 | 90 |

[a]Groups of Balb/C nude mice were given s.c. injections of $2 \times 10^6$ (200 µl) melanoma cells. Mice were monitored weekly for development of palpable tumours. [b]Tumours achieved a diameter of 10 mm, 210 and 330 days post inoculum. [c]Only $1 \times 10^6$ Mel 8 cells were injected.

In cutaneous melanoma, the expression of α3 and αv was not detectable on benign lesions but was expressed on almost all of the metastases. The α4- and α5-subunits were absent on primary cutaneous lesions but were present on seven out of nine and five out of nine of lymph node metastases, respectively, but on only one out of eight skin metastases (Table 4). Expression of αvβ3 was also confined to metastases being detected on five out of nine lymph node and two out of eight skin metastases. In contrast, expression of αvβ5 was higher on the primary lesion (six out of ten naevi, four out of seven VGP) compared with metastases (one out of eight skin metastases); 10 out of 17 vs 1 out of 8 ($P \geq 0.04$, Fisher's exact test).

### DISCUSSION

For malignant cells to metastasize they must decrease their attachment to neighbouring cells. In addition, as maximum motility requires intermediate adhesiveness (Palacek et al, 1997) they may also require reduced adhesion to underlying ECM proteins. This may partly explain why development of breast and colorectal cancer is often associated with reduced or aberrant expression of α2, α3 and α6 (for references see Gui et al, 1997). However, ligation of integrins to the ECM can generate survival signals (reviewed by Meredith and Schwartz, 1996) and, thus, increased expression or de novo expression of specific integrins could also promote cancer.

Cutaneous melanoma is an example of a cancer in which tumour progression correlates with a net gain in several integrins, most notably αvβ3 (Albelda et al, 1990; Danen et al, 1994; 1995; Si and Hersey, 1994; Natali et al, 1997). Although 10% of melanoma occurs in the eye, the majority in the uvea (Shields and Shields, 1992), very little has been documented on the integrins expressed by these tumours. We have therefore compared the expression of integrins by uveal vs cutaneous melanoma cell lines and tissues.

Using flow cytometry (Table 2) and immunoprecipitation (Figure 1) we now show that OM431 is the second uveal melanoma that lacks αvβ3 but expresses αvβ1 a vitronectin/fibronectin receptor (Figures 2 and 3). However, expression of αvβ1 is not universal for all uveal lines as it was not detected in SP6.5 or OCM-1, which instead express αvβ3 (Figure 1).

Analysis of the tumorigenicity of the cell lines (Table 3) revealed that the αvβ3-expressing lines, regardless of uveal or cutaneous origin, were highly tumorigenic, forming xenografts 50–100% of inoculated animals. In contrast the αvβ1-positive lines were either poorly or non-tumorigenic. In addition, using flow cytometry we have measured expression of αvβ5, αvβ6 and αvβ8 on the nine cutaneous melanoma cell lines already examined for αvβ3 expression (Marshall et al, 1991). Together with the data reported here, we have found that formation of subcutaneous xenografts by 17 human (cutaneous and uveal) melanoma lines correlates with an αvβ3-positive, αvβ1-negative phenotype. Thus, our data may suggest that loss of αvβ1 by the VUP and OM431 lines may promote xenograft formation. We have found no correlation between tumorigenicity and expression of αvβ5 or αvβ8 (αvβ6 was not expressed by melanoma cell lines; Marshall and Hart, 1996).

In a recent study, Natali and colleagues (1997) failed to detect any αv-integrins on eight uveal melanomas. However, our analysis of uveal melanoma clinical material confirmed a previous report (ten Berge et al, 1993) that primary uveal melanomas appear to be αvβ3 negative, αvβ5 positive. However, these workers also showed that two of three metastases expressed αvβ3, which we did not observe in our own series. Using the antibodies SZ.21 (anti-β3) and 4B7 (anti-β1), which as reported here work on paraffin-embedded material, only 1 out of 15 uveal melanoma metastases were β3-positive, whereas 8 out of 15 were β1 positive. Thus, unlike cutaneous melanoma, we found no positive correlation between expression of αvβ3 and uveal melanoma metastases.

**Table 4** Immunohistochemical analysis of integrin expression by uveal and cutaneous melanomas

| | Benign Naevus[a] | Radial Growth Phase[a] | Vertical Growth Phase[a] | Nodal Metastasis | Skin Metastasis | Uveal Primary Melanoma | Uveal[c] Metastasis |
|---|---|---|---|---|---|---|---|
| α2[b] | 4/21 | 0/9 | 1/8 | 3/6 | 2/8 | 2/7 | – |
| α3 | 0/21 | 1/9 | 4/8 | 7/9 | 6/8 | 13/13 | – |
| α4 | 0/21 | 0/9 | 0/8 | 7/9 | 1/8 | 0/13 | – |
| α5 | 0/9 | 0/9 | 0/8 | 5/9 | 1/8 | 0/13 | – |
| αvβ3 | 0/21 | 1/9 | 1/8 | 5/9 | 2/8 | 0/13 | – |
| αvβ5 | 6/10 | – | 4/7 | – | 1/6 | 11/12 | – |
| β1 | – | – | – | – | – | – | 8/15 |
| β3 | – | – | – | – | – | – | 1/15 |

[a]The cutaneous melanoma tumours were assessed for histological stage. [b]Frozen cryostat sections were thawed, fixed in acetone (10 minutes at –20°C), air-dried and labelled with antibodies to α2 (P1E6, 1:100), α3 (J143, 1:100), α4 (P4G9, 1:100), α5 (P1D6, 1:100), α6 (GOH3, 1:100), αv (13C2, undiluted supernatant), αvβ3 (23C6, undiluted supernatant), αvβ5 (P3G2, 1:100), β1 (4B7, undiluted supernatant) and β3 (SZ.21; 1:100) [c]Paraffin-embedded material.

We detected integrin αvβ3 on five of nine lymph node and two of eight skin metastases (Table 4), although it should be noted that Natali et al (1997) did not note a difference in expression of this integrin between these types of metastases. In addition unlike previous reports (Albelda et al, 1990; Si and Hersey, 1994), only one out of the eight vertical growth phase lesions was found to be αvβ3 positive. Expression of αvβ5 was higher in the primary lesions (six out of ten naevi, four out of seven VGP) than on the metastases (one out of six skin metastases) in agreement with the data of Danen and colleagues (1995). However, although most metastases from cutaneous melanoma had increased levels of αv integrins, this was not always accounted for by a commensurate increase in either αvβ5 or αvβ3 (Table 4), suggesting that non-β3 αv-integrins were up-regulated.

Like others, we found that α3β1 (Natali et al, 1993), α4β1(Schadendorf et al, 1993), and α5β1 (Danen et al, 1994) show an increased expression on more advanced stages of cutaneous melanoma; particularly on the metastases (Table 4). It may be significant that expression of α4 and α5 was detected on seven out of nine and five out of nine lymph node metastases, respectively, but only on one out of eight skin metastases (Table 4). These data could suggest that expression of these integrins may increase the propensity of melanoma cells to colonize lymph nodes partly, perhaps, by using α4β1 to adhere to VCAM-1 (Mould et al, 1994).

The observation by several groups (Albelda et al, 1990; Danen et al, 1994; 1995; Si and Hersey, 1994; Natali et al, 1997) and ourselves that αvβ3 expression is increased in the later stages of cutaneous melanoma is consistent with this heterodimer having an active role in malignancy. Several functions have been ascribed to αvβ3 that may contribute to such a mechanism. Thus, it has been reported that αvβ3 may cause retention of melanoma cells in lymph nodes through binding to lymph node vitronectin (Nip et al, 1992), whereas ligation of αvβ3 has resulted in increased expression of the metalloproteinase MMP2 (72 kDa type IV collagenase) (Seftor et al, 1992). Recently, Brooks and colleagues (1996) have reported that αvβ3 bound to, and thus located, MMP2 at the surface of invasive cells. Moreover, αvβ3, which is not normally a receptor for interstitial collagen, binds to denatured (for example collagenase-digested) collagen type I and in doing so may provide survival signals to melanoma cells (Montgomery et al, 1994). Thus, in addition to its role as a major adhesive and migratory integrin (Marshall and Hart, 1996), αvβ3 may have other functions during melanoma development.

In conclusion, an αvβ3-positive, αvβ1-negative phenotype is associated with the capacity of cutaneous or uveal melanoma cell lines to form xenografts in nude mice. However, in clinical material, although αvβ3 was expressed by > 50% nodal metastases, the majority of uveal melanoma metastases and cutaneous melanoma skin metastases lacked detectable αvβ3, suggesting that expression of this integrin may not be a prerequisite for formation of either of these melanoma lesions.

### ACKNOWLEDGEMENT

Grateful thanks to Dr. Nigel Kirkham for the supply of melanoma tissue.

### REFERENCES

Albelda SM, Mette SA, Elder DE, Stewart R, Damjanovch L, Herlyn M and Buck CA (1990) Integrin distribution in malignant melanoma: Association of the β3 subunit with tumor progression. *Cancer Res* **50**: 6757–6764

Boukerche H, Berthier-Vergnes O, Dore JF, Leung LLK and McGregor JL (1989) A monoclonal antibody (LYP18) directed against blood platelet glycoprotein IIb/IIIa complex inhibits human melanoma growth *in vivo*. *Blood* **74**: 909–912

Boukerche H, Benchaibi M, Berthier-Vergnes O, Lizard G, Bailly M and McGregor JL (1994) Two human melanoma cell-line variants with enhanced *in vivo* tumour growth and metastatic capacity do not express the β3 integrin subunit. *Eur J Biochem* **220**: 485–491

Brooks P, Stromblad S, Sanders L, Von Schlasa T, Aimes R, Stetler-Stevenson W, Quigley J and Cheresh DA (1996) Localization of matrix-metalloproteinase MMP-2 to the surface of invasive cells by interaction with integrin αvβ3. *Cell* **85**: 683–693

Carter WG, Wayner EA, Bouchard TS and Kaur P (1990) The role of integrins α2β1 and α3β1 in cell–cell and cell–substrate adhesion of human epidermal cells. *J. Cell Biol.* **110**: 1387–1404.

Cheresh, DA and Spiro RC (1987) Biosynthetic and functional properties of an Arg-Gly-Asp-directed receptor involved in human melanoma cell attachment to vitronectin, fibrinogen and von Willebrand factor. *J Biol Chem* **262**: 17703–17711

Danen EHJ, Ten Berge PJM, Van Muijen GNP, Van Thofgrootenboer AE, Brocker EB and Ruiter DJ (1994) Emergence of α5β1 fibronectin receptor and αvβ3 vitronectin-receptor expression in melanocytic tumour progression. *Histopathology* **24**: 249–256

Danen EHJ, Jansen KFJ, Van Kraats AA, Cornelissen MHA, Ruiter DJ and Muijen GNP (1995) αv-integrins in human melanoma: Gain of αvβ3 and loss of αvβ5 are related to tumour progression *in situ* but not to metastatic capacity of cell lines in nude mice. *Int J Cancer* **61**: 491–496

Davies J, Warwick J, Totty N, Philip R, Helfrich M and Horton M (1989) The osteoclast functional antigen, implicated in the regulation of bone resorption is biochemically related to the vitronectin receptor. *J Cell Biol* **109**: 1817–1826

Felding-Habermann B, Mueller BM, Romerdahl CA and Cheresh DA (1992) Involvement of integrin αv gene expression in human melanoma tumourigenicity. *J Clin Invest* **89**: 2018–2022

Fradet Y, Coedon-Cardo C, Thomsen T, Daly ME, Whitmore WF, Lloyd KO, Melamed MR and Old LJ (1984) Cell surface antigens of human bladder cancer defined by mouse monoclonal antibodies. *Proc Natl Acad Sci USA* **81**: 224–228

Gui GPH, Puddefoot JR, Vinson GP, Wells CA and Carpenter R (1997) Altered cell-matrix contact: a pre-requisite for breast cancer metastasis? *Br J Cancer* **75**: 623–633

Hynes RO (1992) Integrins: versatility, modulation, and signaling in cell adhesion. *Cell* **69**: 11–25

Klein CE, Steinmayer T, Kaufmann D, Weber L and Brocker E-B (1991) Identification of a melanoma progression antigen as integrin VLA-2. *J Invest Dermatol* **96**: 281–284

Kramer RH, Vu M, Cheng Y-F and Ramos DM (1991) Integrin expression in malignant melanoma. *Cancer Metas Rev* **10**: 49–59

Marshall JF and Hart IR (1996) The role of αv-integrins in tumour progression and metastasis. *Sem Cancer Biol* **7**: 129–138.

Marshall JF, Nesbitt SA, Helfrich MH, Horton MA, Polakova K and Hart IR (1991) Integrin expression in human melanoma cell lines: Heterogeneity of vitronectin receptor composition and function. *Int J Cancer* **49**: 924–931

Marshall JF, Rutherford DC, McCartney ACE, Mitjans F, Goodman SL and Hart IR (1995) αvβ1 is a receptor for vitronectin and fibrinogen and acts with α5β1 to mediate spreading on fibronectin. *J Cell Sci* **108**: 1227–1238

Mastrangelo MJ, Baker AR and Katz HR (1985) Cutaneous Melanoma. In *Cancer: Principles and Practise of Oncology*, DeVita VT Jr, Hellman S and Rosenberg SA (eds), pp. 1371–1422. JB Lippincott: Philadelphia

Meredith JE Jr and Schwartz MA (1997) Integrins, adhesion and apoptosis. *Trends Cell Biol* **7**: 146–150

Mitjans J, Sander D, Adan J, Sutter A, Martinez JM, Jaggle C-S, Moyano JM, Kreysch H-G, Piulats J and Goodman SL (1995) An anti-αv-integrin that blocks integrin function inhibits the development of a human melanoma in nude mice. *J Cell Sci* **108**: 2825–2838

Montgomery AMP, Reisfeld RA and Cheresh DA (1994) Integrin αvβ3 rescues melanoma cells from apoptosis in three-dimensional dermal collagen. *Proc Natl Acad Sci USA* **91**: 8856–8860

Mould P, Askari J, Craig S, Garrat A, Clements J and Humphries M (1994) Integrin α4β1-mediated melanoma cell adhesion and migration on vascular cell adhesion molecule-1 (VCAM-1) and the alternatively spliced IIICS region of fibronectin. *J Biol Chem* **269**: 27224–27230

Natali PG, Nicotra MR, Cavaliere R, Giannarelli D and Bigotti A (1991) Tumor progression in human malignant melanoma is associated with changes in α6/β1 laminin receptor. *Int J Cancer* **49**: 168–172

 © Cancer Research Campaign 1998

Natali PG, Nicotra MR, Bartolazzi A, Cavaliere R and Bigotti A (1993) Integrin expression in cutaneous malignant melanoma: association of the $\alpha 3\beta 1$ heterodimer with tumour progression. *Int J Cancer* **54**: 68–72

Natali PG, Hamby CV, Felding-Habermann B, Liang B, Nicotra MR, Di Filippo F, Giannarelli D, Temponi M and Ferrone S (1997) Clinical significance of $\alpha v\beta 3$ integrin and intercellular adhesion molecule-1 expression in cutaneous malignant melanoma lesions. *Cancer Res* **57**: 1554–1560

Nip J, Shibata H, Loskutoff DJ, Cheresh DA and Brodt P (1992) Human melanoma cells derived from lymphatic metastases use integrin $\alpha v\beta 3$ to adhere to lymph node vitronectin. *J Clin Invest* **90**: 1406–1413

Nishimura SL, Sheppard D and Pytela R (1994) Integrin $\alpha v\beta 8$: Interaction with vitronectin and functional divergence of the $\beta 8$ cytoplasmic domain. *J Biol Chem* **269**: 28708–28715

Palacek S, Loftus J, Ginsberg M, Lauffenberger D and Horwitz A (1997) Integrin-ligand binding properties govern cell migration speed through cell-substratum adhesiveness. *Nature* **385**: 537–540

Pellegrini R, Bazzini P, Tossi E, Tagliabue E, Conforti G, Dejana E, Menard S and Colanghi MI (1992) Production and characterization of two monoclonals directed against the integrin $\beta 1$ chain. *Tumori* **78**: 1–4

Schadendorf D, Gawlik C, Haney U, Ostmeier H, Suter L and Czarnetzki BM. (1993) Tumour progression and metastatic behaviour *in vivo* correlates with integrin expression on melanocytic tumours. *J Pathol* **170**: 429–434

Seftor REB, Seftor EA, Gehlsen KR, Stetler-Stevenson WG, Brown PD, Ruoslahti E and Hendrix MJC (1992) Role of the $\alpha v\beta 3$ integrin in human melanoma cell invasion. *Proc Natl Acad Sci USA* **89**: 1557–1561

Shields JA and Shields C (1992) *Intraocular Tumors: A Text and Atlas*, WB Saunders: Philadelphia

Si Z and Hersey P (1994) Immunohistological examination of the relationship between metastatic potential and expression of adhesion molecules and 'selectins' on melanoma cells. *Pathology* **26**: 6–15

Sonnenberg A, Janssen H, Hogervorst F, Calafat J and Hilgers J (1987) A complex of platelet glycoproteins Ic and IIa identified by a rat monoclonal antibody. *J Biol Chem* **262**: 10376–10383

ten Berge PJM, Danen EHJ, Van Muijen GNP, Jager MJ and Ruiter DJ (1993) Integrin expression in uveal melanoma differs from cutaneous melanoma. *Invest Ophthalmol Vis Sci* **34**: 3635–3640

Wayner EA, Carter WG, Piotrowicz RS and Kunicki TJ (1988) The function of multiple extracellular matrix receptors in mediating cell adhesion to extracellular matrix: preparation of monoclonal antibodies to the fibronectin receptor that specifically inhibit cell adhesion to fibronectin and react with platelet glycoproteins Ic-IIa. *J Cell Biol* **107**: 1881–1891

Wayner EA, Garcia-Pardo A, Humphries MJ, McDonald JA and Carter WG (1989) Identification and characterisation of the T lymphocyte adhesion receptor for an alternative cell attachment domain (CS1) in plasma fibronectin. *J Cell Biol* **109**: 1321–1330

Wayner EA, Orlando RA and Cheresh DA (1991) Integrins $\alpha v\beta 3$ and $\alpha v\beta 5$ contribute to cell attachment to vitronectin but differentially distribute on the cell surface. *J Cell Biol* **113**: 919–929

Weinacker A, Chen A, Agrez M, Cone RI, Nishimura S, Wayner E, Pytela R and Sheppard D (1994) The role of integrin $\alpha v\beta 6$ in cell attachment to fibronectin – heterologous expression of intact and secreted forms of the receptor. *J Biol Chem* **269**: 6940–6948

© Cancer Research Campaign 1998